# Eco-Friendly Production of AuNPs and Their Impact on the Oil Oxidative Stability

**DOI:** 10.3390/nano11071668

**Published:** 2021-06-25

**Authors:** Flávio S. Michels, Pablo J. Gonçalves, Valter A. Nascimento, Samuel L. Oliveira, Heberton Wender, Anderson R. L. Caires

**Affiliations:** 1 Optics and Photonics Group, Institute of Physics, Federal University of Mato Grosso do Sul, Campo Grande 79070-900, Brazil; samuel.oliveira@ufms.br; 2 Nano & Photon Research Group, Laboratory of Nanomaterials and Applied Nanotechnology (LNNA), Institute of Physics, Federal University of Mato Grosso do Sul, Campo Grande 79070-900, Brazil; heberton.wender@ufms.br; 3 Instituto de Física, Universidade Federal de Goiás, Goiânia 74690-900, Brazil; pablo@ufg.br; 4 Laboratory of Spectroscopy and Bioinformatics Applied to Biodiversity and Health, Faculty of Medicine, Federal University of Mato Grosso do Sul, Campo Grande 79070-900, Brazil; valter.aragao@ufms.br

**Keywords:** gold nanoparticle, vegetable oil, sunflower, oxidative stability, Rancimat

## Abstract

Vegetable oils have been used for different applications and, more recently, as an active host medium to obtain nanoparticles for employment in bionanotechnological applications. Nevertheless, oils are very susceptible to oxidation during production, storage, and transportation because of their chemical composition. Consequently, any modification in their production must be accompanied by an analysis of the oxidative stability. In this study, naked and biocompatible gold nanoparticles (AuNPs) were grown on sunflower oil during sputtering deposition using different deposition times. Size and morphology were determined by transmission electron microscopy (TEM) and their concentrations were found by inductively coupled plasma-optical emission spectroscopy (ICP-OES). Rancimat^®^ method was employed to evaluate the AuNPs influence on the oxidative stability of the vegetable oil. Well-dispersed quasi-spherical NPs were produced with a mean diameter in the 2.9–3.7 nm range and they were concentration-dependent on the deposition time. A concentration of about 11 mg/L, 38 mg/L, and 225 mg/L of AuNPs was obtained for a deposition time of 5 min, 15 min, and 30 min, respectively. The results also revealed that AuNPs negatively affected the oxidative stability of the sunflower oil and exponentially reduced the induction period (IP) with the increase in AuNPs content. IP reductions of 63%, 77%, and 81% were determined for the AuNPs containing samples at 11 mg/L, 38 mg/L, and 225 mg/L. For the first time, it is reported that naked AuNPs promote the rapid degradation of vegetable oil and this points out the need for attention relative to the quality of vegetable oils used to host metal nanoparticles.

## 1. Introduction

Engineered nanoparticles have been applied for different purposes, such as intelligent drug delivery [1,2,3,4,5] and cosmetics [6,7]. Nanoparticles (NPs) can be produced by controlling and manipulating matter, breaking larger particles, or regulating the assembly processes [8]. The chemical composition, stability, morphology, and modulation of the NPs’ surface strictly impact their physical-chemical characteristics and applications [9,10,11].

In cosmetics, nanomaterials are promising technological alternatives for the development of innovative cosmetic formulations [12]. NPs have been widely used in sunscreens, deodorants, makeup, anti-aging care, hair, and oral products [13,14,15,16]. Nanoformulations containing gold nanoparticles (AuNPs) associated with vegetable oils have received much attention because they can enhance permeation through the outermost layer of the skin, which enables, for instance, a transdermal delivery of AuNPs [17]. The permeation efficiency of nanoformulations can be modulated by NPs’ characteristics, such as shape, charge, and functionalization [18,19].

Different technological approaches have been developed to synthesize stable nanosystems and, among them, methods using vegetable oils have stood out. Vegetable oils have been used to design multifunctional nanostructured carriers with multiple health benefits [20,21,22,23]. These oils possess several advantages over synthetic compounds because of their constituents, such as triglycerides, fatty acids, phenolics, and carotenoids. They present antioxidant, anti-inflammatory, and antimicrobial activities and further protects the skin against reactive oxygen species and synergistically improving, e.g., the photoprotective properties of sunscreens [24,25,26]. Moreover, the synthesis of NPs using vegetable oils is considered an environmentally friendly chemical approach [27].

Nowadays, the search for eco-friendly routes has received considerable attention and sputtering has proven to be a clean and valuable method to obtain stable, biocompatible, and naked metal NPs in vegetable oils [28]. Sputtering is a physical method able to produce metallic nanoparticles directly in low vapor pressure liquids [10,11,27,29,30,31]. In this technique, atoms and clusters of atoms may be ejected from the sputtered target in a controlled atmosphere and directly deposited onto the liquid without using chemical stabilizers or toxic reagents, which avoids waste generation; therefore, this method results in a clean, one-step, and simple synthesis process [11,32]. From the physical and chemical point of view, the nucleation and growth of NPs onto liquids by sputtering is governed by different parameters, such as liquid/gas surface chemical composition, liquid substrate coordination ability, discharge voltage, working pressure, liquid substrate temperature, and, among others, sputtered atoms’ kinetic energy. For a detailed discussion, readers are referred to a recently published review article [11]. In summary, both the chemistry of the liquid substrate and the physical parameters of the sputtering process itself may be used to tune the final size of the naked colloidal NPs produced. In the case of using synthetic or vegetable oils for producing biocompatible metal NPs through sputtering deposition, Wender et al. (2011) reported for the first time that the underlining mechanism for controlling the formation of thin films or nanoparticles was by simply controlling the oil surface coordination ability and the sputtering discharge voltage [28] where thin films (nanoparticles) were predominant for low (high) coordinating oils and low (high) discharge voltages. In fact, the discharge voltage directly correlates with the atom diffusion rate at the oil surface and, when it is relatively low, the dominant parameter for nanoparticle formation is the surface coordination ability of the oils. That is why thin films are sometimes produced instead of colloidal nanoparticles [33,34]. Therefore, for the obtention of biocompatible NPs, it is highly recommended to use oils with a satisfactory coordination ability (presence of functional groups and/or unsaturated aliphatic chains). In addition, for high diffusion rates (high discharge voltages), a balance between the physical and the chemical parameters needs to be considered when understanding the NPs formation processes.

The biocompatibility of AuNPs has been extensively studied in human cells [35], which is very important for biomedical and cosmetic applications. However, there is an increasing concern regarding their potential cytotoxicity, which depends on the shape, size, concentration, capping agent, host medium, and biological substrate (human cells, cancer cells, bacteria, etc.) [36,37]. Consequently, attention must be paid to these parameters and their relation to the production of biocompatible nanoparticles.

The oxidative stability is an important quality parameter of vegetable oils, which is straightly connected to, e.g., the shelf life. Moreover, storage conditions related to illumination, temperature, humidity, and contaminants can accelerate oxidation [38,39]. Although AuNPs have been proposed to prolong oil preservation, studies have shown that metals in vegetable oils speed up oxidative processes [40,41,42,43]. Nevertheless, the effects of metallic NPs on the oxidative stability of vegetable oils have not been investigated yet. The present work reports an eco-friendly route to produce, for the first time, naked and biocompatible AuNPs by sputtering deposition onto sunflower oil and investigates the impact of AuNPs on the oxidative stability of sunflower oil.

## 2. Materials and Methods

### 2.1. Vegetable Oil

Sunflower oil (Liza^®^) was used as a medium to produce AuNPs. The sunflower oil was chosen because it oxidizes in a relatively short time scale and this renders it an excellent vegetable oil model for accelerated degradation studies [44].

### 2.2. Production of AuNPs

The AuNPs deposition was performed using a sputtering chamber (Desk III, Denton Vacuum LLC, Moorestown, NJ, USA) with a gold target. The amount of 2 mL of sunflower oil was added to a glass dish of 5 cm diameter and placed 12 cm from the target surface. The chamber was evacuated to around 160 mTorr, followed by argon addition to the chamber up to a working pressure of 330 mTorr. Then, the AuNPs were produced applying a current of 41 mA for three fixed deposition times (5, 15, and 30 min).

### 2.3. Characterization of AuNPs by TEM and EDS

The formation of AuNPs was investigated using a 200 kV Tecnai G2 (FEI, Eindhoven, The Netherlands) transmission electron microscope (TEM) and energy dispersive spectroscopy (EDS). The samples were prepared by sonicating the colloidal solution under ultrasound for 10 min and then a thin layer of the liquid containing the NPs using a 3 mm diameter Cu hoop dipped in the sample was collected. This thin layer was screened on the surface of a holey carbon copper grid. After drying for 24 h, the grids were analyzed in the microscope. The mean size of the NPs was determined by measuring 500 particles found in the grid, which were assumed to be spherical.

### 2.4. Quantification of AuNPs by ICP-OES

Inductively coupled plasma optical emission spectroscopy (ICP-OES) quantified the AuNPs concentration deposited in the sunflower oil samples. An ICP-OES iCAP 6300 Duo (Thermo Fisher Scientific, Bremen, Germany), with the axial and radial view, and a simultaneous detector CID (Charge Injection Device were used to check the procedure’s accuracy. Commercial purity argon 99.999% (White Martins-Praxair, Campo Grande, Brazil) was used to purge the optics, plasma generation, nebulizer, and auxiliary gas. All determinations from ICP OES were carried out in plasma axial view under the following operating conditions: 1350 W RF power, 12 L/min plasma gas flow rate, 0.75 L/m nebulizer gas flow, 50 rpm analysis pump rate, and 30 s integration times. The Au emission line at 242.795 nm was monitored.

All working solutions were prepared using ultrapure water (18 M U cm, Milli-Q^®^, Millipore, Bedford, MA, USA) and nitric acid (65%, Merck, Darmstadt, Germany) and hydrogen peroxide (35%, Merck, Darmstadt, Germany). Single-element stock solutions containing 1000 mg/L of Au (SpecSol, São Paulo, Brazil) were used to prepare standard reference solutions and to carry out spike experiments.

The samples (0.5 mL) were weighed in a PTFE digestion vessel. Concentrated HNO_3_ (2 mL) and H_2_O_2_ (1 mL) were added to the vessel. The decomposition of the samples was carried out in a microwave digestion system SpeedWave (Berghof, Königsee, Germany). The samples were submitted to the step heating procedure exhibited in Table 1. After digestion, the volume was adjusted to 20 mL to perform the quantification.

For quantitative analysis, calibration curves were built using different concentrations of standard Au (0.001, 0.0026, 0.005, 0.01, 0.025, 0.05, 0.1, 0.25, 0.5, 1.0, 2.0, and 4.0 mg/L). The limit of detection (LOD) and quantification (LOQ) were calculated according to IUPAC recommendations in terms of the standard deviation of the baseline (Sbl, *n* = 20). The LOD (=3 * Sbl/m) achieved was 0.0006 mg/L, while the LOQ (=10 * Sbl/m) was 0.002 mg/L, where m is the slope of the calibration curve.

### 2.5. Oxidative Stability

The oxidative stability of the samples was determined by the Rancimat method using the 893 Professional Biodiesel Rancimat (Metrohm AG, Herisau, Switzerland). Analyses were carried out by heating 3.0 g of the sample at 110 °C under an air-flow of 10 L/h according to the European standard EN14112 [45]. The induction period (IP) was obtained from the second derivate of the electrical conductivity versus heating time.

### 2.6. Fatty Acid Composition Determination

The fatty acid content was determined by gas chromatography (GC) with a flame ionization detector (FID) 6890 N (Agilent, Santa Clara, CA, USA) using the following conditions: injector and detector temperature at 225 °C and 285 °C, respectively. Hydrogen flow of 40 mL/min, air flow rate of 450 mL/min, helium flow rate of 40 mL/min, injection volume of 1.0 μL, and column (HP-88 100 m × 0.250 mm). The initial oven temperature program was: 160 °C for 3 min, ramp from 160 to 190 °C (heating rate of 3 °C/min) and from 190 to 230 °C (heating rate of 6 °C/min), and then maintained at 230 °C for 12 min. The identification of the fatty acid peaks was performed by comparing the relative retention time of existing peaks in the sample with the relative retention time of methyl esters mix pattern fatty acids (Supelco FAME C8-C22, 99% purity). Relative retention time was calculated by contaminating the samples and the standard mix with an internal standard (Methyl undecanoate). The methyl esters content was determined based on peak areas.

## 3. Results and Discussions

**Figure 1:**
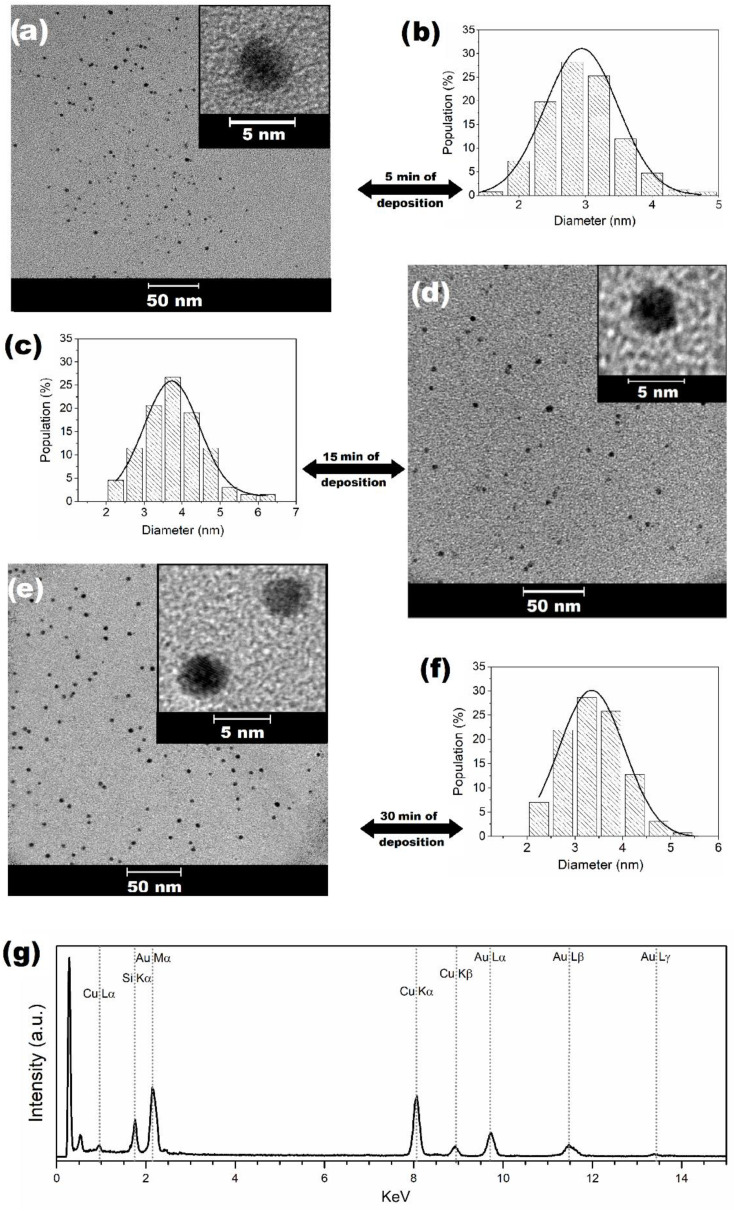
TEM images of AuNPs in the sunflower oil subjected to (**a**) 5 min, (**d**) 15 min, and (**e**) 30 min of Au deposition time and their respective histograms of diameter distribution (**b**,**c**,**f**). Representative EDS spectrum (**g**) of the formed AuNPs in the sunflower vegetable oil during 5.0 min of Au deposition.

Figure 1 shows the TEM images of the naked AuNPs growth directly in sunflower oil by sputtering deposition. Quasi-spherical particles were obtained for all deposition times and so the particle morphology was time-independent (Figure 1a,d,e). The formation of AuNPs was also confirmed by the emergence of the localized surface plasmon resonance (LSPR) absorption band in the green range (Figure S1, Supplementary Material), as expected for spherical AuNPs. Moreover, the AuNPs presented a mean diameter of (2.9 ± 0.5), (3.7 ± 0.7), and (3.4 ± 0.7) nm for the deposition time of 5, 15, and 30 min, respectively (Figure 1b,c,f). These findings revealed that the mean diameters and standard derivation (sigma) were independent of the deposition time. The representative EDS spectrum of the 5 min sample shows the Mα, Lα, Lβ, and Lγ emission lines of the Au, which confirms the chemical composition of the AuNPs (Figure 1g). It is also possible to visualize the Cu and Si lines, where the Cu signal arises from the microscopy grid and the Si from the oil storage glass.

ICP-OES was used to investigate and quantify the AuNPs concentration deposited in the sunflower oil samples for the different sputtering deposition times, Table 2. As expected, a concentration increase was determined as a function of the deposition time. The higher the deposition time, the higher the concentration of Au in the obtained colloidal samples. It is also notable that the Au concentration does not follow a linear trend with the sputtering deposition time. This is likely a result of the heating effects in the target in high deposition times, which requires further investigation.

It is noteworthy to mention that the surface coordination ability of the liquid substrate, viscosity, and the diffusivity of the incoming atoms at the liquid surface are reported among the most important parameters for describing nucleation and the growth of nanoparticles by sputtering deposition on liquids [28,32]. In some cases, thin films may be produced instead of nanoparticles [28]. As all depositions were performed with the same sputtering experimental settings (applied voltage and current at the target) and liquid substrate (sunflower oil), different nanoparticle sizes were not expected. To further obtain insights on the AuNPs formation mechanism by sputtering onto the sunflower oil, the fatty acid composition of the oil was studied, Table 3. As a result, the composition of the sunflower oil is mainly dominated by long unsaturated chain fatty acids, namely, the linoleic and oleic acids, which is as expected [46]. The linoleic fatty acid contains 18 carbons and 2 unsaturations in its chain. Meanwhile, the oleic acid also has a long chain of 18 carbons but with 1 unsaturation. Both acids possess COOH functional groups and unsaturations that may coordinate the incoming Au atoms and/or clusters—by direct chemical bonding or steric effects, respectively—at the liquid/gas interface during the sputtering deposition, which decreases the diffusivity of the metallic atoms on the oil surface and controlling the NPs nucleation process [27,28]. Once the formed nuclei obtain sufficient size to break the liquid surface tension, it is expected that the NPs growth process will occur at the liquid oil phase [27]. This hypothesis is strengthened by the fact that the agglomeration of particles was not observed by TEM, which is expected if the growth occurs at the surface of the liquid, as in the case of silicone oil [47] and caprylic/capric base triglyceride oil [28]. In this last step, the long-chain containing fatty acids protect the individual AuNPs of coalescence by the well-known steric stabilization effect [48,49,50], which finally explains the small size distribution and excellent dispersity of the samples without the formation of the agglomerated regions. These results are evidence for the high potential for biomedical applications, in general, of this naked and biocompatible AuNPs colloidal solution. However, literature is scarce on the oxidative stability of metal NPs containing oils.

**Figure 2:**
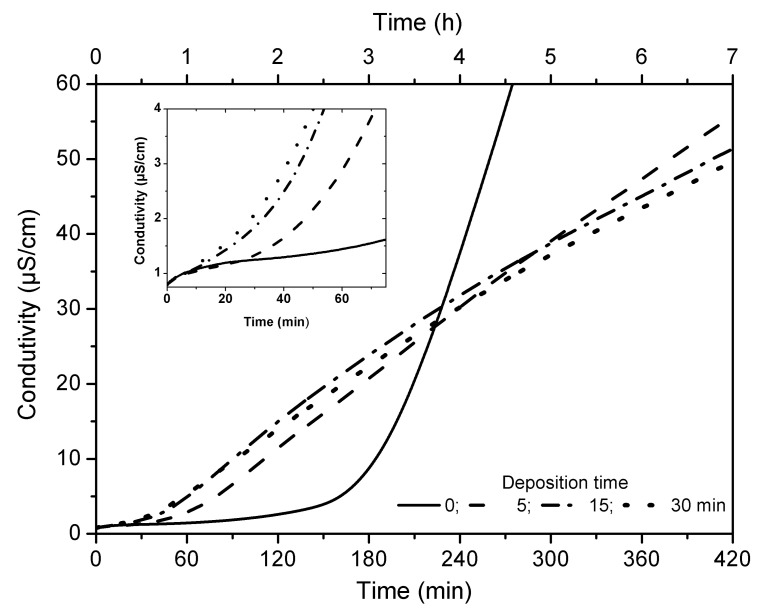
Electrical conductivity as a function of heating time of sunflower oil for different sputtering deposition times.

**Figure 3:**
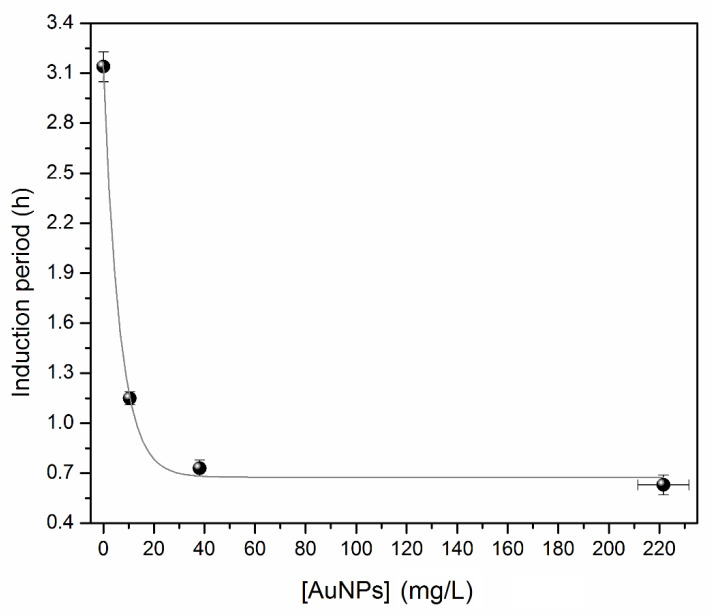
Induction period versus Au content in the vegetable oil.

In order to investigate the stability of the sunflower oil as the host medium for the AuNPs, the oxidative stability of the oil was investigated in detail. Figure 2 shows the electrical conductivity values as a function of heating time. A fast rise of the conductivity in the samples containing AuNPs compared to the one without NPs (solid line in Figure 2) can be observed. The difference is highlighted by the induction period (IP) found for the precursor oil and the oil hosts exposed to 5 min, 15 min, and 30 min of deposition time: (3.14 ± 0.09), (1.15 ± 0.04), (0.73 ± 0.05), and (0.63 ± 0.06) h, respectively. Figure 3 shows an exponential decrease in *IP* as a function of Au concentration, which is described by Equation (1) with R^2^ = 0.9968.

(1) $$IP=0.68+2.46e^{-\left[AuNPs\right]/6.4}.$$

These results are evidence that AuNPs negatively impact the oxidative stability of sunflower oil by accelerating its degradation in a dependent manner on the AuNPs concentration.

The accelerated oxidative degradation of the vegetable oil promoted by the AuNPs may be associated with their capacity for producing reactive oxygen species (ROS) [51,52,53,54,55,56]. It is known that the metal accelerates the oxidation of vegetable oils due to ROS generation [43,57,58]. Although there is vast literature on metal and metal-oxide effects on oxidative stability of vegetable oils, the impact of NPs on vegetable oil stability has not been reported yet.

Therefore, these findings highlight the need for studies on the potential impact of NPs on vegetable oils used as a host medium for the design of beneficial products containing multifunctional and biocompatible nanostructured carriers; this is mainly due to the fact that NPs could severely affect the product’s shelf life.

## 4. Conclusions

Stable naked AuNPs of around 3 nm diameter were produced using an eco-friendly method based on the use of sunflower vegetable oil and the sputtering technique. Sunflower oil was revealed as an excellent host medium for obtaining small AuNPs (<5 nm) because of its COOH functional groups and its long and unsaturated alkyl fatty acid chains. The results also indicate that AuNPs negatively affected the oxidative stability of the sunflower oil and accelerates its oxidation. The IP significantly decreased, presenting a reduction of 63%, 77%, and 81% for AuNPs content of 11 mg/L, 38 mg/L, and 225 mg/L. Consequently, AuNPs promote an accelerated oxidative degradation of the vegetal oil and so precautions must be taken to assure the quality control of vegetable oils used to host AuNPs in bionanotechnological products.

## Figures and Tables

**Table 1:** Heating program used in the microwave digestion of the samples.

Step	Temperature (°C)	Time (Min)	Pressure (Bar)
1	120	10	30
2	180	15	30
3	250	10	30
4	50	10	20

**Table 2:** Au concentration in sunflower oil for different deposition times and respective accuracy (RSD).

Deposition Time (Min)	Concentration (mg/L)	%RSD
5.0	10.52 ± 0.09	0.87
15.0	38.07 ± 0.14	0.37
30.0	224.58 ± 10.06	4.54

**Table 3:** Fatty acid composition in sunflower oil.

Fatty Acids	%
Palmitic, C16:0	5.9 ± 0.3 ^1^
Palmitoleic, C16:1	0.2 ± 0.0
Stearic, C18:0	3.2 ± 0.1
Oleic, C18:1	36.8 ± 0.3
Linoleic, C18:2	49.5 ± 0.1
Linolenic, C18:3	0.2 ± 0.0
Arachidic, C20:0	0.2 ± 0.0
Cis-11-Eicosenoic, C20:1	0.2 ± 0.0
Ducosanoic, C22:0	0.6 ± 0.1
∑ Saturated	9.9
∑ Unsaturated	86.9

^1^ The values are means ± standard derivations.

## Data Availability

Data are available upon request from the authors.

## Supplementary Materials

The following are available online at https://www.mdpi.com/article/10.3390/nano11071668/s1, Figure S1: UV-vis absorption spectra of sunflower oil as function of Au deposition time. INSET. Absorbance at 531 nm, related to the plasmon resonance of AuNPs, as function of the deposition time.
