# Supplementary material for: Eco-Friendly Production of AuNPs and Their Impact on the Oil Oxidative Stability
Source: Nanomaterials (Basel). 2021 Jun 25;11(7):1668. doi: 10.3390/nano11071668 (PMC8308139; doi:10.3390/nano11071668)
Supplement: Supplementary file 1 [file nanomaterials-11-01668-s001.zip › nanomaterials-1214914-supplementary.pdf]

SUPPLEMENTARY MATERIAL

Article

Eco-Friendly Production of AuNPs and Their Impact on the Oil Oxidative Stability

Flávio S. Michels [1,2,*], Pablo J. Gonçalves [3], Valter A. Nascimento [4], Samuel L. Oliveira [1], Heberton Wender [2] and Anderson R. L. Caires [1,*]

[1] Optics and Photonics Group, Institute of Physics, Federal University of Mato Grosso do Sul, Campo Grande 79070-900, MS, Brazil; samuel.oliveira@ufms.br
[2] Nano & Photon Research Group, Laboratory of Nanomaterials and Applied Nanotechnology (LNNA), Institute of Physics, Federal University of Mato Grosso do Sul, Campo Grande 79070-900, MS, Brazil; heberton.wender@ufms.br
[3] Instituto de Física, Universidade Federal de Goiás, Goiânia 74.690-900, GO, Brazil; pablo@ufg.br
[4] Laboratory of Spectroscopy and Bioinformatics Applied to Biodiversity and Health, Faculty of Medicine, Federal University of Mato Grosso do Sul, Campo Grande 79070-900, MS, Brazil; valter.aragao@ufms.br
* Correspondence: flavio.michels@ufms.br (F.S.M.); anderson.caires@ufms.br (A.R.L.C.)

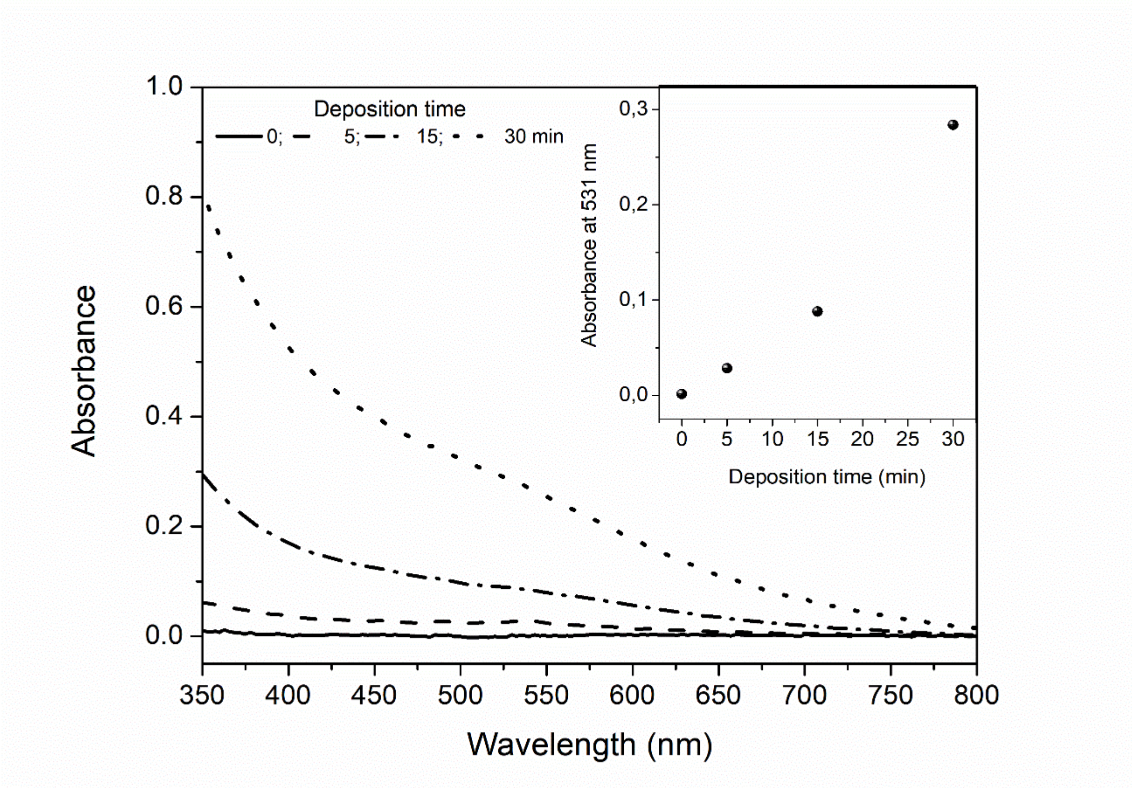

Figure S1. UV-vis absorption spectra of sunflower oil as function of Au deposition time. INSET. Absorbance at 531 nm, related to the plasmon resonance of AuNPs, as function of the deposition time.
